# Attracting Green Consumers as a Basis for Creating Sustainable Marketing Strategy on the Organic Market—Relevance for Sustainable Agriculture Business Development

**DOI:** 10.3390/foods9111552

**Published:** 2020-10-27

**Authors:** Boban Melović, Dragana Cirović, Tamara Backovic-Vulić, Branislav Dudić, Katarina Gubiniova

**Affiliations:** 1 Faculty of Economics, University of Montenegro, 81000 Podgorica, Montenegro; bobanm@ucg.ac.me (B.M.); dcirovic@ucg.ac.me (D.C.); tassabacc@ucg.ac.me (T.B.-V.); 2 Faculty of Management, Comenius University in Bratislava, 82005 Bratislava, Slovakia; katarina.gubiniova@fm.uniba.sk; 3 Faculty of Economics and Engineering Management, University Business Academy, 21000 Novi Sad, Serbia

**Keywords:** food choice, organic production, green consumers, agriculture business, sustainable consumption

## Abstract

The aim of this paper was to analyze the main factors that affect green consumers’ choice regarding the purchase of organic agriculture products. The data collected through a survey of 559 green consumers were analyzed using explanatory factor analysis, the Relative Importance Index, and logistic regression. The results point out eleven main factors related to the offerings on the organic agriculture market that predominantly drive green consumers’ purchasing decisions. The Relative Importance Index identified health benefits that stem from a specific way of production as the main purchasing motive. This was also confirmed by the results of logistic regression, which showed that a respondent who buys organic agricultural products on a daily basis is approximately 71.5% less likely to disagree with the claim that organic products are healthier than non-organic, compared to a consumer who purchases organic products several times a week or month. However, as these benefits cannot be empirically confirmed, green consumers look for official labels on the product packaging. In order to assure the product quality, more than half of them find out very important information about producers: whether they have a product quality certificate (69.5%), how many years they are engaged in production (56.2%), and whether they have specific product packaging (54.9%). The Relative Importance Index also revealed that the main purchasing barriers that consumers face are scarce offerings and an insufficient development of the distribution channel, which were ranked in first and second place. The price as a barrier is of less importance. About 30.8% of the respondents are willing to pay up to 20% higher prices for organic food compared to conventional food, while 39.4% of them would pay even up to 40% higher prices. Based on the given results, there are clear suggestions for creating a sustainable marketing strategy for organic agriculture products as the main prerequisite for an increase of healthy food choices and fostering the future development of organic agriculture businesses at the local and global levels.

## 1. Introduction

Raising pressure to achieve further economic growth and at the same time resolve the negative consequences that intensive industrial development has caused, such as the disturbed balance of the ecosystem, stressed economic dependency on non-renewable energy sources, and raising inequalities throughout the society, has fostered decision-makers to implement the sustainability concept into their business strategy. This concept implies finding different approaches to economic development and social prosperity, which is based on the rational use of renewable resources, preservation of human health, and environmental protection [1,2]. However, although the sustainable development concept highlights the main goals that should be achieved, its implementation cannot be standardized and depends on the specificities of each economic activity, market demand, available resources, and perceived costs of its implementation [3]. Therefore, sustainable marketing has a central role in implementing sustainable practices in economic activities.

Sustainable marketing is based on the postulates of sustainable development and incorporates not only economic concerns but also social and environmental responsibilities in business activities. It implies the production of sustainable products, defining prices that reflect the sustainability effort of producers, the development of sustainable marketing channels, and promotion that is not focused only on the product itself but also on raising awareness about the importance of sustainable consumption [4,5]. However, a key to successful sustainable marketing is creating an effective strategy that will deliver these values to the end consumers [4,5,6].

Although the context of sustainable marketing can be viewed through various aspects, such as the state of the environment or climate change, this research focuses on the characteristics of sustainable production and consumption viewed through the prism of organic products. Thus, the concept of a sustainable marketing strategy is considered from a development perspective. In other words, the research combines an analysis of the market segment of green consumers with their selection and appropriate positioning in order to appropriately place organic products, which are seen as the basis for creating a sustainable marketing strategy. Considering the characteristics of the sustainability concept and sustainable marketing, organic agriculture production is recognized as the main driver of sustainable economic development in many countries, especially in rural areas.

Total retail sales in the organic food market reached 97 billion euros in 2018. The market growth was noted in most of the countries in the world, and in several of them, it was in the double digits [7]. However, it still forms a very small part of the total food market sales. Therefore, further efforts should be made in order to develop local markets of organic agricultural products and increase the total number of organic buyers, so that all the social benefits it offers can be achieved.

Although organic products are being sold at premium prices, a more important obstacle to stimulating demand that organic producers face is the fact that the characteristics of organic products that differentiate them from conventional ones are intangible and cannot be confirmed through their consumption. Therefore, producers and other decision-makers should create a different marketing strategy that will highlight the sustainability implications of consuming these products [3,8]. A sustainable marketing strategy on the organic food market incorporates an economic, social, and environmental dimension of business, and it is organized through four main elements of the sustainable marketing mix—sustainable organic product, price, sustainable marketing channels, and promotion [4,5,6]. However, a sustainable marketing strategy should create a competitive advantage on the organic market through raising awareness about the importance of sustainable consumption and by targeting consumers who find values of economic, social, and environmental responsibility very important. In order to achieve that, it is necessary to understand the key factors that shape consumers’ perception of the offerings on the organic market.

Organic production is recognized as a basis for future sustainable development in many countries, including Montenegro—a country in which this research has been conducted. It is a small country characterized by a clean environment and preserved natural resources, which has great potential for engagement in organic food production. In order to maintain a healthy natural environment, the government of Montenegro adopted the Declaration on the Ecological State of Montenegro, which defined the strategic commitment of a country to adopt and apply the highest standards and norms in the field of environmental protection and economic development on the principles of ecologically sustainable system [9]. Hence, fostering organic production is in line with this previously stated goal. However, the organic food market is underdeveloped, it is characterized by scarce domestic offerings, and consumers face a lot of ambiguities regarding the concept of the organic production. According to the data retrieved from the Monteorganica (the institution in charge of running the organic certification procedure), in Montenegro, there are 319 organic producers [10]. They are predominantly engaged in organic plant production (270 of them). However, considering the potential that Montenegro has for fostering organic food production, several different supporting programs aimed at producers have been implemented that offer educational and financial benefits [11]. Financial support has been found to be very important, as it provides domestic producers with loans and funds from different projects (retrieved from several European funds). The funds received from projects were more favorable compared to domestic loans in terms of interest rates and in terms of grants that went up to 60% [11,12].

The organic products in Montenegro are being sold at prices that are on average 30%–50% higher compared to the prices of conventional ones [11]. Most of them are being sold in the supermarkets, specialized organic stores, as well as in hotels and restaurants that often sell these products as a part of the tourist offer. However, the most significant amount of organic food products are those that consumers purchase directly from producers they know, in order to assure that the products they buy are truly organic.

All these stated characteristics are common not only to the Western Balkan countries but to most of the other countries where the organic food market is developing [13,14,15,16]. Hence, it can be expected that the results obtained in this research can be considered important for understanding the consumers’ choice regarding organic food products in other countries, too.

Previous research on this topic investigated consumers’ perception of the main characteristics of the organic products, their prices, distribution channels, and promotion, and identified factors that can shape that perception. However, there is a lack of research about the relative strength of the impact of each of these factors. Additionally, most of this research focuses on consumers in general, without investigating the differences regarding their perception and existing awareness about the importance of sustainable consumption.

In order to fill in these gaps, this research aims to investigate the key factors related to the sustainable marketing mix that shape the perception and attitudes of green consumers toward organic food products and therefore affect its consumption. Based on the research results, this paper will give insight into how certain aspects of the sustainable marketing mix elements should be organized in order to create a successful sustainable marketing strategy for the organic agricultural products, with the aim of attracting especially green consumers as a segment that highly values the sustainable dimension of products.

In addition to the theoretical application, this paper has a significant practical contribution. Green consumers are the main drivers of sustainable consumption and can have a significant impact on raising awareness about its importance throughout society. Understanding the relative strength of the impact that certain factors have on their perception of the offerings on the organic food market is the main prerequisite for creating a successful marketing strategy that will highlight the impact of positive ones and minimize the impact of negative ones. Therefore, it would enable producers and other decision-makers to better adapt the offerings on the organic market to the green consumers’ expectations while emphasizing the sustainable dimension of these products. Additionally, understanding the main factors that drive the consumption of this specific consumer segment gives insight into the main aspects of the sustainable marketing mix on the organic food market that should be highlighted when targeting other consumer segments and raising awareness toward sustainable consumption. Creating a successful marketing strategy would increase the demand for sustainable products and improve the competitiveness of organic producers compared to conventional domestic and foreign competitors. This way, it would foster agricultural business development, especially in rural areas, and it would foster overall further organic market development. Therefore, it would make a very important contribution to solving some of the most important problems, such as environmental pollution, overdependence on non-renewable energy sources, poverty, social inequalities, food waste, and especially the economic underdevelopment of rural areas that most of the countries face nowadays.

## 2. Literature Review

### 2.1. Theoretical Background

Although there is no unique definition of green consumers, most of the research identifies them as consumers whose consumption is significantly affected by their environmental and social concerns [17,18]. These consumers believe that their actions have a significant impact on the environment and they contribute to its preservation through purchasing green products [19]. Additionally, these consumers often purchase green products due to their social image, which makes them appealing for social identity-based marketing strategies [20]. However, although many green consumers state that they have very positive attitudes toward green products, most of them buy them only occasionally, which is known as an attitude–behavior gap [21]. Therefore, there is still little known about how green consumers actually make their purchasing decisions and the key factors that shape their consumption.

Earlier studies tried to identify the main demographic characteristics of green consumers. The research of Diamantopoulos et al. and Jain et al. revealed than green consumers are mostly younger or middle-aged, with a high level of education and a high level of income [22,23]. On the other hand, a study of Shiel et al. obtained opposite results, showing that age is the only demographic characteristic of green consumers that plays an important role in green products purchase due to its relation to environmental knowledge and environmental concerns [18]. Similar results were obtained in a study of Feil et al., which revealed that socioeconomic and demographic factors do not have a significant impact on green consumers’ purchasing behavior [24]. Additionally, a segmentation analysis revealed that green consumers’ purchase and their willingness to pay premium prices for green products are influenced by the social altruism and the level of their concerns on environmental pollution, as well as by the level of their personal responsibility to contribute to environmental preservation and fostering green initiatives. However, segmentation analysis failed to give more precise insight into the main factors that shape their buying behavior [17,19].

In order to identify the main drivers of green consumers’ purchases in general, including the purchases in the organic food market, further research has been conducted. The research of Banytė et al. [17] as well as a study by Smith and Paladino [25] revealed that all green consumers are concerned with the solution of environmental issues. Therefore, environmental knowledge and concern for the future are very important factors that lead to forming positive attitudes toward green and organic food [18,26]. Similar results were obtained in the research of Adhitiya and Astuti [27], revealing that the social value of organic products has a significant positive impact on green consumer behavior, while the functional, epistemic, emotional, and conditional values of these products is of much less importance. Additionally, a study of Lobo and Greenland [28] revealed that collectivism and long-term orientation as dimensions of culture also have a positive influence on the purchase of green and organic products. Additionally, the research of Seegebarth et al. [29] confirmed that cultural dimensions affect the consumers’ attitudes toward green products and especially their recommendation behavior. This is in line with previous research considering the fact that a preserved environment and human well-being are the common resources, and these goals can be achieved only in the long-term. However, a study of Troudi and Bouyoucef [26] obtained the opposite results, revealing that long-term orientation does not have a significant effect on green consumers’ attitudes toward green and organic products. The opposite results were also found in a study of McEachern and McClean [30], suggesting that the self-interest-centered purchasing motives have a much stronger effect compared to altruistic motives of green purchase.

However, protection of the environment is very important but yet not always the primary motive for the purchase of green and organic products, as some green consumers search for these types of products mostly due to health concerns [16,17]. Health is a very important reason for organic product purchases, as green consumers consider these products as safe, healthy and free from genetically modified organisms [19,31]. On the other hand, a study of Aagerup and Nilsson [21] revealed that social pressure is often the main driver of green products purchases, including organic food. However, it should be noted that the personality of a consumer moderates the response to given circumstances and the social impact of making purchasing decisions [21].

The organic food products meet all previously analyzed requirements that green consumers seek—they are safe, healthy, their production is sustainable, and it enables protection of the environment and all the natural resources. Organic agriculture production is an innovative system that enables the production of safe and healthy products while preserving the environment and natural resources. It is based on principles of sustainability, whose implementation results in significant benefits for the whole society [32]. It is well adapted to the local conditions, which is why it is recognized as one of the key drivers of sustainable economic development in many countries, especially in the case of rural areas [32,33,34]. Organic production implies avoiding the use of fertilizers, pesticides, animal drugs, and food additives that can have a negative impact on human health, which results in the production of safe products. It minimizes the negative impact on the environment and attains ecological balance through establishing production processes adapted to the local conditions and natural resources without disrupting it [32,33]. Additionally, it is built on relations that ensure respecting the highest ethical values, therefore increasing the welfare not only of the organic market participants but of every member of the society. However, total sales on the organic food market make up only a small percentage of total food market sales, which highlights two main obstacles to the further development of organic agriculture. It is evident that only a small number of loyal organic consumers buy most of the total sold organic agricultural products. A more important obstacle is the fact that the biggest organic producers are placed in less developed countries in Asia and Africa, while almost 90% of organic agricultural products are being sold in the USA and Europe [7,35]. This points out the serious imbalance between demand and supply at the local market level. In addition, it emphasizes the significance of a better understanding of key elements of a sustainable marketing strategy and sustainable marketing mix that should be pointed out in order to foster green consumers’ purchase on the organic food market.

### 2.2. Factors Influencing Green Consumers’ Purchase of Organic Agriculture Products

Previous research in this field mostly investigated consumers in general and their response to certain elements of the offerings in the organic food market. Considering the fact that organic products have added value compared to conventional products that is not visible and cannot be experimentally confirmed, many authors pointed out the significance of creating specific packaging and its impact on purchasing decisions [36,37,38]. A study of Aschemann-Witzel revealed that placing nutritional and health claims on organic food packaging has a very significant positive impact on regular organic buyers, while its impact on occasional buyers is of less importance [39]. The results supporting these findings were especially obtained in studies conducted in Central and Eastern Europe, suggesting health concerns as main motives for organic food purchase. A study of Bryla [13] revealed that Polish consumers perceive these products as healthier compared to conventional ones, as well as of higher quality. The health concerns are also confirmed as the main motive for purchasing organic food products in Romania [40]. The same results are obtained in a study conducted in Western Balkan countries, indicating that main consumers’ motives for buying organic food products are health concerns and environmental protection [14,15]. A study of of von Meyer-Höfer et al. [16] investigated whether there are significant differences regarding expectations toward labeled organic products between developed (Germany and UK) and emerging organic markets (Spain and Czech Republic). The results suggested that consumers expect labeled organic products to be free of chemical pesticides, mineral fertilizers, and genetically modified organisms. These were the top three expectations in each observed country, which also emphasizes the importance of health concerns on purchasing decisions and thus the importance of trust in organic labels and claims highlighted on the products’ packaging.

The findings on the importance of trust in organic labels were confirmed in research by Enax and Lu et al. [19,41] and pointed out the impossibility of distinguishing organic products from conventional ones as one of the main obstacles that green consumers face when buying these products. Additionally, a study of Teng and Wang [42] revealed that increasing consumers’ trust through organic certification and labeling is an important prerequisite for purchase. Similar results were obtained in the research of Papista and Dimitriadis [43] that revealed the importance of creating an organic brand for attracting green consumers. These results show that creating confidence and long-term relations with green consumers enables them to express their personality and altruism through organic or other green brand purchases, which has a significant impact on their final behavioral outcomes and making a purchasing decision. The importance of brand image was also confirmed by a study of Puelles et al. [44], which revealed that consumers consider a producer’s brand as of better quality compared to the distributor’s brand. On the other hand, a study of van Herper [45] pointed out that any kind of packaging has a negative impact on organic food sales, as it limits the choices that consumers can make when buying these products. These findings were supported also in a study conducted by Petrescu et al. [46] in Romania, which revealed that non-labeled products (from farmers’ markets consisting of self-produced products) are considered as more “organic” compared to certified ones. Additionally, the research of the Irish Food Board revealed that Irish consumers value organic products based on their benefits stemming from specific production processes, while additional features of these products (such as packaging) are of far less importance [47]. These results are also confirmed in a study of Hoffmann and Schlicht [48]. Therefore, the analysis of previous research revealed that there is still a lack of knowledge of which aspects of organic food products should be emphasized in order to create a successful sustainable marketing strategy when targeting green consumers.

Price is regarded as one of the main obstacles when buying organic food products [19,49,50,51]. The research of Renko et al. [14], as well as the research of Melovic et al. [15] and Ham et al. [50] revealed that this barrier is particularly important for consumers in the Western Balkan countries. Additionally, this is an especially significant obstacle for occasional green buyers and non-core organic consumers, which is why the reduction of regular prices is very important for stimulating the purchase of these consumers [52,53]. Similar results were obtained in a study of Marian et al. [54] suggesting that although the promotional cut of prices does not have an impact on organic food purchase, the reduction of regular prices has a significant influence on stimulating regular green consumers’ purchases, especially when there is another clue regarding the high quality of the product besides premium prices. On the other hand, a study of Troudi and Bouyoucef revealed that green prices do not have a significant negative impact on the consumers’ attitude toward green products [26]. These findings were also confirmed in a study of Adhitiya and Astuti [27] as well as in the research of Diallo et al. [55]. Considering the contradicting results obtained in previous research, it is still unclear how green consumers form attitudes toward green products’ prices and which aspects of the offerings should be highlighted when creating a sustainable marketing strategy in order to justify the premium green prices of these types of products.

The poor development of distribution channels also represents a very important obstacle to further organic market growth, and removing this barrier is very important for encouraging non-core green consumers to increase their green purchases. Core green consumers are well informed about the organic production concept and the availability of the organic offer. They seek shops that offer a wider range of certified organic food products, so they mostly make their purchases in specialized green and organic stores and are ready to make an extra effort when buying green products. However, this is not the case with non-core green consumers. Therefore, including organic products in conventional stores’ offerings is one of the main prerequisites for attracting this type of consumer [56]. The research of Islam et al. [57] and Henryks et al. [58] pointed out that although organic products have premium prices, offering these products in conventional stores does not influence consumers’ perception of the store’s price level, but it has a positive influence on consumers’ perception of the social dimension of the store image. Additionally, these studies [57,58] revealed that the development of distributors’ organic brands would create significant benefits for retailers as well as for green consumers and foster organic market development. These findings were supported by a study of Puelles et al. [44] suggesting that although producers’ brands are perceived as of better quality, distributors’ green brands offer an excellent price–quality ratio. These brands have lower prices compared to producers’ green brands, which makes them an attractive option for non-core and price-sensitive green consumers. On the other hand, purchasing directly from farmers represents a very important channel of distribution in developing and emerging organic markets, such as those in Romania, Estonia, and Western Balkan Countries, which makes consumers sure that the products they buy are truly organic [13,16,59]. Therefore, the previous research failed to answer the question of whether green consumers consider a lack of organic food products in conventional stores as a significant obstacle, or their inability to find a wider range of organic food products in any type of store is a more important barrier in the purchasing process. Additionally, it is still unclear whether a green label or other similar characteristics has a significant impact on attracting green consumers when buying organic food products.

Taking into account the health benefits of the consumption of organic food products, previous research indicated that the promotion of these products should be based on pointing out health claims on packaging and through other promotion channels, as well as on educating green consumers on how to differentiate them from conventional ones [60,61]. Similar results were obtained Enax and Weber in their study, pointing out the significance of placing a recognizable organic label on the product packaging [41]. Additionally, a study of Kereklas et al. [62] revealed that promotional messages containing both altruistic and egoistic claims are an effective tool for attracting green consumers. However, the previous research did not focus on measuring the effectiveness of different promotion channels while delivering these messages. Although Internet-based channels, such as social media, can be a very effective way of reaching a large number of green consumers [63,64,65], it is still unclear what media are the most suitable for promoting organic food products for attracting this segment of consumers.

Considering the literature gap regarding different aspects of the sustainable marketing mix and sustainable marketing strategy on the organic food market that is addressed above, the aim of this paper is to investigate what features of the offer should be emphasized when targeting green organic consumers. Understanding the main factors that shape green consumer purchases is the main prerequisite for creating a successful sustainable strategy for attracting these consumers and enabling local agriculture business development. The authors have consulted previous research in the field in order to identify potential factors that have a significant influence on green consumers’ attitudes toward organic agriculture products and therefore determine a sustainable marketing strategy that organic producers should implement. Some of them are given in Appendix A of this paper.

The results of previous research identified many different factors, yet they failed to identify which of them has a prevalent role in attracting green consumers on the organic food market. Therefore, the first research question has been formulated as follows.

RQ1: What are the key factors that affect green consumers’ perception of the organic agriculture products on offer?

In order to foster the development of organic agriculture businesses and local organic markets, the producers should create a marketing strategy based on pointing out the factors that have a positive impact on the green consumers’ perception of organic agriculture products, while at the same time reducing the potential purchasing barriers that green consumers face. Therefore, the second research question is formulated as follows.

RQ2: What are the key marketing strategy determinants that organic agriculture businesses should adopt to attract green consumers?

**Figure 1:**
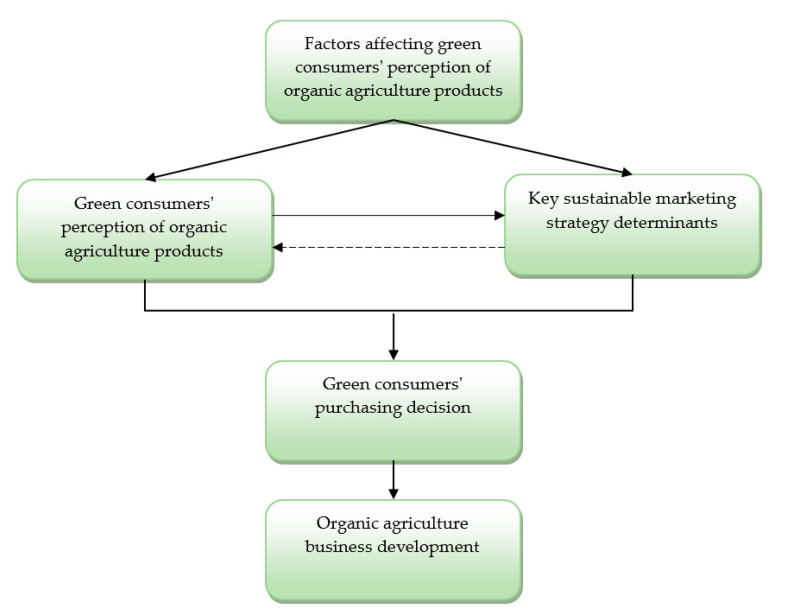
Conceptual model of the research (source: authors).

The conceptual model of the study is formulated based on the given research questions, and it is given below in Figure 1.

The conceptual model of the research is based on the causality of three main constructs: green consumers’ perception of organic products, factors that affect it, and the appropriate sustainable marketing strategy that organic agriculture businesses should adopt. Although many factors influence green consumers’ perception of organic agriculture products, they do not have the same significance. Based on the given research questions, this study aims to identify factors that have a prevalent role in that process. The identification of those factors will enable a better understanding of how green consumers perceive organic agriculture products and what they consider as very important for making a purchasing decision. Through an understanding of how green consumers perceive organic products, as well as the main factors that affect it, producers will be able to create a sustainable marketing strategy that points out the importance of positive factors and diminishes the impact of negative ones. The implementation of such a marketing strategy would attract green consumers and foster their purchase of organic products, which is one of the main prerequisites for the future organic agriculture business development. However, although consumers’ perceptions affect the suitability of the given sustainable marketing strategy, it should be noted that the applied marketing strategy also affects and changes the perception of green consumers toward the organic food offerings. Therefore, the purchasing decision is the final result of the interaction between those two concepts, as the given conceptual model shows. The obtained results of the research based on this conceptual model can provide significant guidelines for marketing managers and organic producers, pointing out how the main elements of the sustainable marketing strategy should be organized, in order to increase sales of the organic food products and foster agriculture business development on sustainable grounds.

## 3. Methodology and Sample

Considering the formulated research questions, the conceptual model, results, and conclusions of previous studies in this field, as well as previous experience of the authors, the questionnaire was developed. In order to test its validity, a pilot survey was conducted on 20 respondents. Based on their recommendations, the final form of the questionnaire was developed, and it consisted of 20 questions. It comprised two types of questions: multiple-choice questions and a five-point Likert scale. The questions were grouped into several sections in order to include the key factors that can have an important impact on the organic food purchase. In order to include every important aspect of the organic offer in the questionnaire, the authors developed each set of the questions considering the obtained results from previous research in the field. More precisely, the first group comprised multiple-choice questions regarding the demographic characteristics of respondents, such as their gender, age, education, and income, as some of the previous research indicated those characteristics as important for making purchasing decisions [17,18,19,21,22,23]. The questions from the second group were related to green consumers’ perception of organic agriculture products, as well as the main motives and barriers to purchase. Based on the results of previous research regarding this topic [17,18,25,26,27,28], these questions comprised a set of given potential motives for organic food purchase (such as impact on health, nutritional value, environment protection, etc.), as well as a set of given potential purchasing barriers (such as premium prices, scarce offerings, bad appearance of organic products, inability to find them in stores, etc.). These questions were given in the form of a set of statements, and the responses were measured using a 5-point Likert scale. Additionally, this set also contained a multiple-choice question regarding the most important incentive that would encourage potential consumers to purchase organic agriculture products. Considering the importance of product distribution and the previous research on this issue [14,35,51], the third set comprised multiple-choice questions related to the distribution channels of these products in order to identify the types of stores where respondents purchase organic agriculture products and which kind of stores they perceive as the best for selling these products. Additionally, it comprised a question in the form of a set of statements regarding preferred ways of organic food purchase, and the responses were measured also using a 5-point Likert scale. Considering the results of previous research that pointed out the impact of premium prices on the organic food purchase [19,49,50,51], the fourth set of multiple-choice questions was related to price in order to identify how much higher prices green consumers are willing to pay for organic agriculture products and to what extent price reduction can be an incentive for more frequent purchases. Finally, taking into account its importance confirmed by the previous research [26,61,62,63,64,65], the fifth set of questions was related to promotion, in order to identify what kind of information about organic products green consumers search for and what media they consider a reliable source of information. Additionally, it included questions regarding other significant information (such as specific product label, specific claims, certain information about the producer, etc.) that should be pointed out using promotion instruments in order to encourage organic food purchases. Each set of questions was aimed at better understanding the importance and intensity of factors related to the organic offerings that affect green consumers’ purchasing decisions, in order to provide clear suggestions for creating a successful sustainable marketing strategy in the organic agriculture products market as the main prerequisite for the future development of this economic sector.

After the final form of the questionnaire was developed, a survey was pursued in Montenegro in 2019, and it lasted for 3 months. There are several reasons for conducting a survey in the mentioned country. The organic market in Montenegro is still underdeveloped. However, there is a great potential for organic agriculture development in this country, considering the natural resources it has [66]. The improvement of this economic sector is recognized as one of the priorities, which is why various support programs have been developed aimed at fostering local organic agriculture producers [67]. However, according to the authors’ knowledge, only a little research on this topic has been conducted in this country so far to date. Therefore, it is necessary to further investigate the perception and attitudes of consumers in order to point out the direction of the effort of organic producers and other decision-makers that will make the best possible contribution to the development of the organic market. Additionally, the obtained results can be of significant importance for other countries where the organic market is in its early stages of development, and they can serve as a basis for further research on this topic.

In order to identify the green consumers that will take part in the survey, the research was pursued in several food stores in the country that sell organic or other eco-friendly products. There were surveyed consumers who purchased organic or other eco-products and agreed to take part in the research. Considering that the aim of the research is to reach the buyers of green food products, it was appropriate to conduct the research in stores that sell these products. Although the aim might be better achieved if the sampling procedure took place in specialized organic stores, it should be noted that there are only a few stores of this type in the country, selling mostly cereals. Hence, the selection of conventional food stores that sell a wider range of organic and eco-products enabled researches to target a wider range of green consumers. In addition to the range of the organic products, the food stores were selected also based on their size. Considering that there are in total 24 municipalities in Montenegro, the survey was conducted in four largest towns from each region of the country: the northern, central and southern. We chose up to the three largest food stores per city (depending on the city size). Hence, the sampling procedure took place in eight stores in total from the four largest towns in the north of the country, ten stores from the four largest towns located in the central region of the country, while eight stores were chosen from the four largest towns from the south region of the country. Therefore, the sample provided a relatively balanced representation of stores from across the whole country, which led to a stratified random sample.

Regarding the characteristics of the respondents, we did not include any other strata in the sample besides that of whether the consumer purchased organic and/or green products in a store. The reason for this decision is the fact that previous research gave contradictory results toward the impact of the demographic characteristics of respondents on purchasing decisions [18,22,23,24]. Additionally, there is no previous research in Montenegro that investigated the impact of demographic or other characteristics of the respondents on organic food purchases, so there were no available data that could serve as the basis for selecting a stratified sample through targeting the precisely defined strata of the population. Therefore, a stratified convenience sampling methodology was used, and the sample consisted of 559 valid questionnaires. More than half of the respondents (65%) were women, while 35% of them were male. Although the gender was not the basis for the stratification of the sample, the given gender structure is representative, considering the fact that women do the shopping more often than men. The respondents were mostly younger than 40 (74.4%) and had a high school diploma (42%) or university degree (46.9%). The given results of the sample structure can be explained by the fact that it did not comprise all the buyers of the given store; instead, it was only those who purchased organic or other green products. It turned out that those are mostly younger and highly educated consumers. This implies that these consumers are better informed about the concept of organic production and the characteristics of the organic products, which is why they prefer to visit the stores that offer them. Although the given notion should be further explored, it is not the aim of this paper. A more detailed overview of the structure of the sample is given below in Table 1.

Further data analysis was proceded by determining the reliability of the survey using Cronbach’s alpha. The obtained value of this coefficient is 0.787, which is an acceptable value of this indicator in the social sciences. In order to identify the main factors that affect green consumers’ perception of organic agriculture products, we applied multivariate analysis i.e., exploratory factor analysis, the Relative Importance Index (RII), and the logistic regression. Exploratory factor analysis aims at explaining the relationship of many observed variables by a relatively small number of factors. In other words, it enables the identification of the factors that have the strongest influence on the observed variable. In this research, the exploratory factor analysis defined factors of key importance for making the decision to buy organic food products. In order to rank the main motives of the organic food purchase by their importance, as well as to rank the purchasing barriers, we calculated the Relative Importance Index. This index is the mean value of the importance of the factor that gives it weight according to the perception of the respondents. Finally, logistic regression is most commonly used to rank the relative importance of independent variables (i.e., purchasing motives and barriers) and to quantify the effect of their impact.

## 4. Results and Discussion

### 4.1. The Extraction of Key Factors Affecting Green Consumers’ Perception of the Organic Agriculture Products on Offer

In the first part of the research analysis, we obtained factor analysis in order to answer the first research question and identify the key factors that affect green consumers’ perception of the organic agriculture products on offer.

In factor analysis, a pattern of relationships between a large number of variables is sought. Therefore, it begins with determining the correlations of the original variables. In the given analysis, Pearson’s simple correlation coefficient was used, which shows the strength and direction of the connection between the two variables. Correlation matrix analysis showed that it is justified to continue conducting factor analysis because among the analyzed variables, there is a sufficient number of correlation coefficients whose values are greater than 0.3. Although the questionnaire included 22 questions, it should be noted that the questions measured using a 5-point Likert scale consisted of a set of statements, and all of these statements can be considered as a specific factor that fosters or limits the organic food purchase. Therefore, the research included about 40 potentially significant variables, which additionally justifies the application of exploratory factor analysis.

Further analysis identified common factors found in the table of correlation coefficients using the principal components analysis. This method finds groups of variables that have high coefficients within the group and small ones relative to other groups. For further research, we considered the first several main components that have the greatest impact (the largest factor loadings) and cover most of the variability of the data, which is why they represent key identified factors that affect green consumers’ perception toward organic agricultural products. Factor rotation was done using Varimax rotation (which maximizes the sum of the variances of the factor loadings squares) with Keiser normalization. The obtained results are given below in Table 2.

The application of factor analysis defined eleven factors that significantly influence the green consumers’ perception of organic agriculture products and thus their decision to purchase these products. Namely, by applying the method of principal components, eleven factors whose eigenvalues were greater than 1 were extracted. Based on the percentage of variation explained by the extracted factors, the first eleven factors are considered relevant, and they explain 86.881% of the total variations.

In order to assign adequate names to the extracted factors, factor loadings are observed for each variable in order to determine their role and contribution to the definition of the factor structure. Factor loadings greater than 0.50, regardless of its sign, represent moderate to large loadings and show how the variable is related to the factor. Table 3 shows the variables with the significant factor loadings for each identified factor.

The given results of factor analysis show that there are eleven main factors that affect green consumers’ perception of organic agriculture products, and they are named considering the variables that had the biggest contribution to their formation. The first factor represents green consumers’ attitudes toward organic products and is defined by eight variables (whose coefficient values are bolded in Table 2). The second factor, named through the analysis of five variables that are common to it, represents characteristics of organic producers. The third factor was defined by four variables, and it represents distribution barriers. Common to the variables that determined the fourth factor is satisfaction with the food store. The fifth factor represents product packaging and labeling, and it was developed by the joint influence of five variables. The sixth factor was defined by two variables and represents consumer satisfaction with conventional products. The seventh factor represents the ratio of price and consumer income considering the fact that it was defined based on the influence of these two variables. The eighth factor represents the income and level of education of a consumer. The ninth factor represents the impact of the product on health, and it was also created by the influence of two variables. The tenth factor is extracted based on the influence of two variables, and it represents the perception of consumers about the relationship between price and product quality. The last, eleventh factor is related to promotional activities, and it was distinguished by the joint influence of two variables.

Starting from the significance and characteristics of the variables that define them, all identified factors (except income and level of education) can be related to the four basic elements of the offerings on the organic agriculture products market: product, price, distribution channels, or promotion. Five of them are related to organic agriculture products and have a great influence on the consumer perception of those products: green consumers’ attitudes toward this product category; characteristics of the producer; product packaging and labeling; the impact of the products on health; and satisfaction with conventional products. However, the biggest influence is related to green consumers’ attitudes toward these types of products. Such results are not surprising, considering the results of previous research, which shows that consumers most often evaluate organic products based on their objective characteristics and specificities of the production process (such as health safety, nutritional value, environmental protection, animal welfare, etc.) [17,18,19,26,27,28,31,42,43,44,45]. However, since (apart from the taste) none of the listed characteristics by which organic food products differ from conventional ones can be empirically confirmed, consumers are looking for appropriate visible evidence that the given products are truly organic [19,32]. The required evidence can be a recognizable packaging with a prominent certification mark or trust in a well-known and verified manufacturer. Hence, packaging and information about the manufacturer are listed as important factors in the purchase of organic food products, which is in line with previous research [13,19,31,36,37,39,68,69].

Green consumers’ satisfaction with conventional products, as the most important substitutes, appears as a factor that negatively affects the perception of consumers about these products. However, one of the key influences on the formation of this factor is the variable that expresses the opinion of consumers that there is no real difference between organic and conventional food products. This can be explained based on the results of previous research that such an attitude is related to a lack of information about the organic production concept and its total economic and sustainable impact [70,71,72]. Therefore, the given attitude is characteristic of consumers who do not have enough information about the specifics of organic production, and their education and better information are imposed as an effective method of removing this barrier in the further development of the organic food market.

The perception and attitudes of consumers about the prices of organic products are predominantly influenced by two factors: the relationship between prices and consumer income, and the relationship between the price and quality of these products. The analysis of the implications of these factors is very important, taking into account the fact that premium prices are cited as one of the most important barriers (if not the most important) that green consumers face when buying organic agriculture products [19,49,50,51]. The extraction of these two factors in terms of importance confirms that the price as a barrier is also expressed in the Montenegrin market, having in mind the relatively modest purchasing power of the population. However, at the same time, these factors indicate that if consumers consider organic products to be of high quality, they will be willing to pay slightly higher prices for them. At the same time, this emphasizes the importance of additional educating and informing green consumers about all the benefits of organic production, both for them as individuals and for society as a whole, which will justify premium prices considering the high quality and benefits of these products. In addition, it explains the identification of consumer income and education as an important factor that affects organic agriculture products purchase. These results are in line with research conducted by Troudi and Bouyoucef [26] and in line with a study of Adhitiya and Astuti [27], which indicated that premium prices do not lead to negative attitudes of green consumers toward this product category. Additionally, these results clearly indicate that premium prices are not an insurmountable barrier to the wider acceptance of organic food products, which implies that its impact largely depends on consumer information and education about the specifics of organic production systems and the resulting characteristics of organic food products, which will justify premium prices.

Insufficiently developed distribution channels also represent an important barrier when buying this type of product on the Montenegrin market, which is confirmed by the importance of factors that represent distribution barriers and satisfaction with the sales facility. Analysis of the variables that form these two factors shows that due to scarce supply and poor representation in stores, green consumers are not able to buy organic agriculture products because they do not know where to find them or because their purchase requires too much time. In addition, even if they know in which stores these products are being sold, green consumers are not satisfied with the diversity of the offer, nor how these products are exposed and highlighted compared to conventional ones. These results are in line with the previous research [57,58] that points out the poor development of distribution channels as a common barrier to most of the underdeveloped organic food markets.

Regarding the role of a promotion as an instrument of supply, two factors were identified: the promotion of organic food products through the mass media and consumer satisfaction with the information highlighted on the product packaging. Such results are expected, given that the properties of these products that differ from conventional ones cannot be empirically confirmed, which is why it is necessary to provide consumers with an appropriate label in order to facilitate the recognition of this product category [60,61,62,63,64]. Hence, the promotion of these products through the mass media is imposed as a way of informing consumers about the stores where these products are available and about the ways in which they are exposed compared to conventional ones. Additionally, in underdeveloped organic food markets, such as the market in Montenegro, it is expected that consumers are not sufficiently informed about the concept of organic production. Therefore, promotion has a very important role in educating and informing consumers about the characteristics of organic products, the certification process, the available offerings, recognizable labels, and information displayed on the package and other characteristics that could be important in the process of accepting these products.

Finally, considering the fact that there is no available research in Montenegro that investigated the impact of demographic characteristics of consumers on organic food purchases, the factor analysis in this paper included those characteristics. The results indicate that the level of education and level of income are variables that have a significant impact on the organic food consumption. However, these results are not surprising, considering the fact that these products have premium prices, which is why their purchase depends on the purchasing power of the consumers. Additionally, the level of education also affects the decision to buy organic food products in two ways. First of all, the consumers with a higher level of education are usually better informed about the concept of organic production and the benefits it offers. Secondly, these consumers usually have a higher level of income, which increases their purchasing power as an important prerequisite to buying organic products at premium prices.

### 4.2. The Key Marketing Strategy Determinants on the Organic Agriculture Products Market

Although some important conclusions can be derived from the previous results, we applied further analysis about the relative strength and importance of factors that determine the final decision of green consumers related to organic agriculture products purchase in order to answer the second research question (i.e., to make suggestions toward creating a sustainable marketing strategy for selling these products). Hence, the further data analysis included the application of the Relative Importance Index and logistic regression with the aim of measuring the relative strength of given purchasing motives and barriers. These results suggest what motives should be especially pointed out in the marketing strategy of the agriculture producers and the key barriers that must be removed or at least diminished.

Based on the results of previous research [17,18,19,21,25,26,27,28,31], there were eight listed motives of great importance that drive the consumption of these products. Those motives are given in Table 4. The respondents rated the importance of the given factors when making purchasing decisions on a scale from 1 to 5. In order to determine which of them has a dominant role in the purchasing process, we used the Relative Importance Index (RII). The Relative Importance Index (RII) in this research was computed using the following equation [73,74,75]:

RII = (1∙N_A_ + 2∙N_B_ + 3∙N_C_ + 4∙N_D_ + 5∙N_E_)/(5∙N).

In the given equation, 1, 2, 3, 4, and 5 are the Likert scale weightage given to each factor by the respondents; N_A_, N_B_, N_C_, N_D_, and N_E_ are the total numbers of respondents who gave specific weight to the observed factor; 5 is the highest weight and N is the total number of respondents. Table 4 given below shows the ranking of each given factor that has an impact on the organic products purchasing decision.

The results of the Relative Importance Index show that the most important factor in making the decision to buy organic agriculture products is the fact that respondents believe that these products are healthier compared to non-organic ones, while the second factor indicates their willingness to support local producers throughout the purchase. The third most important factor is also related to health and explains that organic products are bought and consumed because they have a positive impact on the prevention and treatment of certain diseases. On the other hand, of the least importance is the factor that indicates that organic products have a higher nutritional value compared to conventional ones.

In order to additionally explain the significance of each factor and the rank it gained, we also applied logistic regression. The dependent variable in logistic regression is the frequency of purchase of respondents, and independent variables are respondents’ attitudes toward the characteristics of organic products stated above as factors that foster purchase. Before the analysis of the model, its quality was examined by testing the null hypothesis that there is no relationship between the dependent and independent variables in logistic regression. The model was tested by comparing the initial value of the logarithm, i.e., the model without an independent variable (which is 959.644) with the final model with an independent variable (which is 502.825). The obtained value of χ^2^ is 456.819 with 62 degrees of freedom, which is significant at the level of 0%. These results show that the model is meaningful and that the null hypothesis cannot be accepted.

The results of logistic regression (given in Appendix B of this paper) suggest that a respondent who buys organic agricultural products on a daily basis is approximately 71.5% less likely to disagree with the claim that organic products are healthier than non-organic compared to a consumer who purchases organic products several times a week or month. In other words, the consumers who buy these products on a daily basis point out to a larger extent the attitude that the organic products are healthier compared to non-organic than a consumer who has a lower frequency of purchases. These consumers are also 67.5% less likely to disagree that organic agriculture products are suitable for health preservation and 36.4% less likely to disagree with the claim that these products are free from GMO and other additives compared to consumers with less purchase frequency. Additionally, they are significantly less likely to disagree with the claims that organic agriculture products have bigger nutritional value, taste better, that their production ensures animal welfare and does not harm the environment, as well as that their purchase provides support to local producers (20.1%, 62.8%, 48%, 58.8%, and 69.9%, respectively). Therefore, the obtained coefficient values in the logistic regression confirm the results of the Relative Importance Index about the rank of given factors fostering organic agriculture products purchase.

The results of the Relative Importance Index and logistic regression are in line with previous research [16,17,19,31] that state health care as one of the most important motives for purchasing organic products. However, it is a bit of a surprising conclusion that environmental protection is ranked in fifth place according to its importance, considering the results of the studies of Smith and Paladino [25], Troudi and Bouyoucef [26], and Adhitiya and Astuti [27] that pointed out environmental concerns as one of the primary motives for green products purchase. However, this can be explained by the fact that Montenegro is an ecological state [9], so the respondents are not exposed to a significant extent to the negative impacts of the environmental pollution.

In addition to the analysis of main motives, we also identified the significance of certain limiting factors that green consumers face when purchasing organic agriculture products. Based on the results of previous research [19,49,50,51,60,61,62,63], we identified eight very important barriers (given in Table 5), and the respondents rated them on a 5-point Likert scale according to their importance. The obtained values of the Relative Importance Index for each of them are also given below in Table 5.

The factor that prevents organic agriculture product purchases with the strongest impact is a limited supply, i.e., an insufficient diversity of these products in the stores. The second most important barrier is the fact that finding organic products is difficult and time-consuming. The third and fourth most important limitations are related to the price, which is considered to be high for customers compared to their level of income or compared to the product quality. On the other hand, the least significant barrier to buying organic agriculture products was the fact that consumers could not see the difference between these types of products compared to conventional ones.

In order to additionally explain the obtained rank of given limiting factors, the logistic regression is applied again. This time, the independent variables were the identified limiting factors (barriers), while the dependent variable was again the purchase frequency. The model was tested by comparing the initial value of the logarithm, i.e., the model without an independent variable (which is 1019.928) with the final model with an independent variable (which is 336.008). The obtained value of χ^2^ is 683.919 with 72 degrees of freedom, which is significant at the level of 0%. These results show that the model is meaningful.

The results of the estimated logistic regression (given in Appendix C of this paper) confirmed that the respondents who buy organic agriculture products on a daily basis are more likely to disagree with given statements regarding purchasing barriers compared to the respondents who buy these products with less purchasing frequency. Therefore, the likelihood that the consumers who purchase organic products on a daily basis will disagree with given statements (compared to consumers with less purchase frequency) is the biggest for barriers related to the availability of the offer and the price: scarce offerings (74.3%); insufficient availability of organic agriculture products in stores, which is why its purchase requires too much time (54.4%); high price compared to the level of income (49.4%) and high price compared to the product quality (49.03%). The likelihood that consumers who purchase organic products on a daily basis will disagree with the rest of the given barriers (compared to consumers with less purchase frequency) is significantly less: lack of information about organic agriculture products (34.71%); inability to find them (27.5%); the worse appearance of organic products compared to non-organic (23.7%); the worse taste of organic products compared to non-organic (22.7%), and the attitude that there is no difference between organic and conventional products (11.85%).

These given results of the Relative Importance Index and logistic regression are in line with previous research [13,19,49,50,56] that state premium prices and poor development of distribution channels as main limiting factors on the organic food market. However, the results also enable a better understanding of these barriers. It points out that although green consumers know where organic agriculture products can be found, they often are not willing to make an extra effort when doing their shopping, which implies the necessity for producers to significantly increase the availability of their products in conventional stores. In addition, the given range of price as a limiting factor suggests that producers should place more effort on justifying premium prices, especially through emphasizing the positive impact of organic agriculture products on health as well as the significance of its purchase for supporting local farmers.

The obtained results of the Relative Importance Index and logistic regression provided the basis for answering the second research question through emphasizing the main motives that should be pointed out when selling organic food products, as well as through pointing out the main barriers that should be removed or at least diminished in order to increase the sales on the organic food market. Further discussion on the practical implications of the results related to the sustainable marketing strategy that producers should adopt is given below in the next section.

## 5. Practical Implications of the Results

Based on the total results of factor analysis, as well as the significance of certain motivating and limiting factors of purchase, it is possible to give clear suggestions to organic producers and other decision-makers in order to create a successful sustainable marketing strategy for attracting green consumers to the organic agriculture market. Namely, green consumers buy organic products thanks to a number of benefits that a specific method of production provides. However, these benefits cannot be empirically confirmed. Therefore, the appropriate labeling and packaging is even more important for organic products compared to conventional ones. The labeling and packaging of conventional products has a role (amongst others) to attract consumers and to differentiate the products of given producers from the competitors’ offerings. On the other hand, the labeling and packaging of organic products has one more very important roles, which is to enable consumers to differentiate these products from conventional ones, considering the fact that its benefits originating from its specific production methods cannot be experientially confirmed. The results of descriptive statistics revealed that almost half of the respondents (40.1%) in Montenegro differentiate organic products from conventional ones based on the official certification mark placed on the product packaging. On the other hand, 35.1% of respondents buy these products directly from producers they are familiar with in order to assure that the food they purchase is really organic. These results show that producers, along with other decision-makers, should place more effort on increasing the recognizability and the reliability of the official organic certification mark in order to enable consumers to differentiate organic products from conventional ones and increase the purchase. In addition to that, pointing out relevant information about the producer is also important. The results of descriptive statistics show that consumers prefer if the producer has the following: an appropriate product quality certificate (69.5%), many years of experience in organic production (56.2%), products with specific product packaging and labels (54.9%), and a wider range of organic products on offer (49.4%). Therefore, the producers should point out this information on the product packaging or use the appropriate instruments of promotion. Finally, producers should also point out the benefits of the organic products purchasing, such as their positive impact on health and support for local producers, considering the fact that these are the main drivers of organic food consumption.

The obtained results also indicate that it is necessary to increase the availability and diversity of these products by the further development of distribution channels such as supermarkets or specialized stores. The results of descriptive statistics reveal that 55.6% of respondents consider that the organic food products should be available in the supermarkets all the time, while 19% of respondents believe that there should be more specialized organic stores. Therefore, the other very important suggestion to the organic producers in Montenegro is to develop their channels of distribution, especially through making arrangements with large retail chains.

However, when creating a sustainable marketing strategy for selling organic agriculture products, special attention needs to be also paid to price and promotion. Although consumers show different degrees of price sensitivity, what they have in common is that they form the final perception of prices of these products based on the assessed price–quality ratio. Hence, producers and other decision-makers must point out in the right way all the benefits of the organic production and consumption of organic agriculture products. If consumers recognize these benefits, they will also be willing to pay premium prices. The fact that the majority of respondents in the sample have average or lower than average incomes confirms that the previous conclusion also applies to price-sensitive consumers, but it also explains the significance of price/income ratio as a purchasing barrier. Additionally, the results of the descriptive statistics reveal that 30.8% of the respondents are willing to pay up to 20% higher prices of organic food compared to conventional food, while 39.4% of them would pay up to 40% higher prices. These results reveal that high prices are not an insurmountable obstacle and that their importance can be significantly reduced by removing other barriers such as poor distribution channel development and scarce offerings. However, in order to justify premium prices, it is necessary to invest more effort in the promotion of organic food products, predominantly relying on the mass media. The results also reveal that 27.4% of respondents consider that the organic food products should be promoted using mass media. Another important promotion instrument is product tasting at fairs (23.6%), while 12.2% of respondents consider that the focus should be placed on educating consumers about all the benefits of the organic production concept and its importance for preserving the environment rather than on promoting organic products using any type of mass media. These results are very important for organic producers when creating their promotional strategy, considering the fact that promotional activities have a very important role in educating and informing consumers about the quality of organic agriculture products and in justifying their premium prices, but also in forming and strengthening the positive attitudes about them compared to conventional products. In addition, promotional activities also inform consumers about the available offerings on the market as well as about the stores where organic agriculture products are available for purchase. In this way, the impact of price and distribution channels as the two main obstacles to the acceptance of these types of products can be removed or at least reduced. At the same time, reducing the impact of these barriers will meet the preconditions for further development of the organic agriculture products market through attracting green consumers and increasing sales volume, which is in line with the efforts of economic policymakers to achieve economic development on sound and sustainable grounds.

## 6. Conclusions

Organic agriculture production is recognized as an innovative system that enables the production of safe and healthy products while preserving the environment and natural resources. It is based on principles of sustainability, whose implementation results in significant benefits for the whole society [32]. Therefore, it has an irreplaceable role in achieving meaningful socioeconomic and ecologically sustainable development. Hence, this paper aimed to provide clear suggestions to organic agriculture producers for creating a successful marketing strategy based on the identification of factors that shape green consumers’ purchasing decisions as a basis for the future development of agriculture business at the local and global level. The data were collected using a survey of 559 green consumers and analyzed using explanatory factor analysis, the Relative Importance Index, and logistic regression. The results of factor analysis identified eleven factors that have a predominant role in the decision-making process of green consumers’ on the organic agriculture products market. Five of them are related to organic products, pointing out the importance of consumers’ attitudes of this product category, as well as ways of their distinction from conventional ones, such as a purchase from a trusted producer or the reliability of product labeling and information placed on product packaging. The results of factor analysis also pointed out the poor representation of organic products in food stores and insufficient development of distribution channels as very significant barriers to further organic agriculture business development and highlighted the necessity of justification of premium prices for this product type. The results of the Relative Importance Index and logistic regression ranked the main fostering and limiting factors of purchase regarding their importance for green consumers, pointing out health concerns and support to local producers as main drivers of their purchase, as well as scarce supply and a lack of distribution channels developments as main barriers. The main theoretical contribution of the paper stems from the fact that the research is conducted in a developing market where the consumers’ perception and attitudes are different compared to the consumers’ beliefs on the developed markets. While previous research investigated the factors affecting organic product purchases, this paper also compares the relative strength of their impact. Finally, through the application of an abductive approach, this paper gives a synthesis of green consumers that prefer organic food products and the creation of a sustainable marketing strategy, therefore making a significant contribution to the existing literature that investigates the green consumers’ behavior and the development of the organic food market.

In addition to the theoretical contribution, this paper provided significant practical implications. The obtained results served as a basis for providing clear suggestions for organic agriculture producers for creating a sustainable marketing strategy for attracting green consumers and selling their products as a key prerequisite for the future development of the agriculture business sector. It also provides suggestions that are applicable for other emerging organic markets due to the common obstacles that mostly characterize them, such as the poor development of channels of distribution and lack of trust in official certification labels.

The main limitation of this paper is related to the fact that the research is conducted only in Montenegro. However, there is a lack of research on this topic in the given country, despite the fact that it is a country that recognized organic production as a basis for the future development of the agriculture sector and the overall development of rural areas, which points out the theoretical and practical contribution of the given research. In addition, the organic food market in Montenegro is still underdeveloped, so the obtained results can serve as a basis for similar research in other countries in which the organic food market is still insufficiently promoted. The additional limitation of this study is related to the fact that the respondents in the sample were green consumers, regardless of their differences in demographic characteristics. In order to overcome the given limitation, future studies on this topic should include the respondents from several countries and observe the differences in the factors that affect their purchasing decisions depending on their demographic or other relevant characteristics, in order to create a marketing strategy adjusted to each significant segment of consumers. However, considering the fact that consumers’ preferences can change over time, similar research should be conducted in each country that recognizes organic agriculture businesses as a substruction of the future economic development of rural areas on healthy and sustainable grounds.

## Figures and Tables

**Table 1:** An overview of the structure of the sample.

Sample Characteristics	Number	%	Sample Characteristics	Number	%
**Gender**			**Organic food purchase frequency per person in the household**		
**Male**	197	35	Every day	116	20.8
**Female**	362	65	At least once a week	239	42.8
			1–3 times a month	204	36.5
**Age structure**			**Average monthly income per person in the household**		
**15–25**	208	37	Less than 500 euros	342	61.2
**26–40**	209	37.4	500–800 euros	153	27.4
**41–60**	100	17.9	800–1300 euros	46	8.2
**More than 60**	42	7.5	More than 1300 euros	18	3.2
**Level of education**					
**Primary school**	5	0.9	Master’s Degree	40	7.2
**Secondary school**	235	42	PhD	17	3
**Bachelor**	262	46.9			

**Table 2:** The extraction of factors using principal components analysis.

Component	Initial Eigenvalues			Rotation Sums of Squared Loadings		
	Total	% of Variance	Cumulative %	Total	% of Variance	Cumulative %
1	10.552	23.449	23.449	7.478	16.617	16.617
2	5.434	12.076	35.525	5.114	11.365	27.982
3	4.863	10.806	46.331	4.608	10.240	38.222
4	3.630	8.067	54.398	3.891	8.647	46.869
5	2.990	6.643	61.041	3.417	7.594	54.463
6	2.574	5.720	66.762	3.134	6.964	61.427
7	2.383	5.295	72.057	2.644	5.876	67.302
8	2.310	5.134	77.191	2.557	5.682	72.985
9	1.735	3.855	81.046	2.351	5.225	78.210
10	1.444	3.209	84.254	2.262	5.026	83.236
11	1.182	2.626	86.881	1.640	3.644	86.881

**Table 3:** Variables with significant factor loadings for each identified factor.

Factor	Variable (Factor Loadings)
Green consumers’ attitudes toward organic agriculture products	Healthier than non-organic (0.835); They are suitable in the prevention and treatment of certain diseases (0.924); Do not contain GMOs, pesticides, antibiotics, and other additives (0.918); Bigger nutritional value compared to non-organic (0.850); Better taste (0.814); Organic production does not harm the environment (0.918); Organic production ensures animal welfare (0.958); The purchase of organic products provides support to local producers (0.810)
Characteristics of organic producers	How important it is if a producer is from Montenegro (0.764); How important it is that the manufacturer has the appropriate product quality certificate (0.586); How important it is for you that the producer has many years of experience in organic production (0.875); How important it is whether the manufacturer has packaging with a specific label (0.743); How important it is whether the producer has a variety of organic products in his offer (0.931)
Distribution barriers	I do not buy organic products in larger quantities because the offer is scarce, not diversified (0.870); I do not buy organic products in larger quantities because they are not sufficiently represented in retail outlets, so buying them requires too much time (0.865); I do not buy organic products in larger quantities because I do not know where to find them (0.902); I do not buy organic products in larger quantities because I do not have enough information about their characteristics (0.561)
Satisfaction with the food store	The importance of diversity of supply (0.641); Satisfaction with the way organic products stand out compared to inorganic ones in retail outlets (0.797)
Product packaging and labeling	The importance of packaging appearance (0.807); The importance of package size (0.806); Importance of information about the producer (0.589); Satisfaction with the look and size of the packaging (0.519); Satisfaction with the design of the label (0.577)
Green consumers’ satisfaction with conventional food products	I do not buy organic products in larger quantities because they taste worse than non-organic ones (0.553); I do not buy organic products in larger quantities because there is no real difference between organic and non-organic products (0.536)
Price/income ratio	I do not buy organic products in larger quantities because of the high price considering my level of income (0.673); The importance of price for purchase (0.609);
Level of income and level of education	Average monthly income (0.849); Level of education (0.856)
The impact of the product on health	The importance of nutritional value for purchase (0.575); The importance of product impact on health (0.579);
Price/quality ratio	I do not buy organic products in larger quantities due to the high price compared to the quality of the products (0.548); Higher price willing to pay for organic compared to non-organic products (0.704);
Information	How satisfied are you with the information highlighted on the product packaging (0.536); Mass media are an important source of information about organic products (0.595)

**Table 4:** The ranking of factors fostering organic products purchase using the Relative Importance Index.

Variables	1	2	3	4	5	RII	Rank
They are healthier	20	23	69	152	295	0.842934	1
Suitable for the prevention and treatment of diseases	24	32	115	171	217	0.787835	3
Do not contain GMO and other harmful substances	31	19	196	169	144	0.734526	7
Higher nutritional value	37	26	197	172	127	0.716637	8
Better taste	13	59	90	208	189	0.779249	4
Protection of the environment	27	31	144	179	178	0.761002	5
Animal welfare	18	38	174	156	173	0.753131	6
Support to local producers	29	24	86	224	196	0.791055	2

**Table 5:** The ranking of factors preventing organic products purchase using the Relative Importance Index.

Variables	1	2	3	4	5	RII	Rank
High prices compared to the level of income	74	129	81	176	99	0.634705	3
High prices compared to product quality	87	164	155	114	39	0.547764	4
Their purchase requires too much time	41	67	152	158	141	0.704114	2
The offer is scarce (not diversified)	34	65	121	237	102	0.710197	1
Taste worse than non-organic	189	157	144	42	27	0.442934	8
Do not know where to find them	113	172	168	81	25	0.504472	6
Worse appearance compared to non-organic	191	137	136	75	20	0.455456	7
No difference compared to non-organic products	198	169	115	39	38	0.438998	9
Do not have information about their characteristics	149	124	141	97	48	0.518068	5

## Appendix A

**Table A1:** Previous research undergrounding the research model and questionnaire development.

References	Sample	Methodology	Object of Research
Khare and Pandey, 2017 [51]	990 consumers in 30 Indian food stores	Confirmatory factor analysis (CFA)	The role of green self-identity and green peer influence on consumers’ perceived trust in retail
McCarthy et al., 2016 [25]	Online peer-based survey of 404 respondents in China	Content analysis, the prohibit model, frequency distribution, and t-test	The determinants of green food and certified organic food purchase
Papista and Dimitriadis, 2019 [31]	A random sample of 848 consumers	Structural equation modeling (SEM)	The impact of confidence, altruism, and self-expression on green brand purchase
Troudi and Bouyoucef, 2020 [21]	A convenience sample of 304 Algerian consumers	Structural equation modeling (SEM)	The impact of green marketing and personal factors on green food purchase
Song et al., 2016 [26]	A non-probability convenience sample of 430 organic food consumers in Malaysia	Structural equation modeling (SEM)	The impact of marketing stimuli on consumers’ attitudes toward organic food
von Meyer et al., 2015 [24]	An online survey of 1180 respondents from four European countries	Mean value analysis and exploratory factor analysis	Consumers’ expectations of labeled organic food products
Ham et al., 2016 [34]	A sample of 411 households in Eastern Croatia	Regression analysis	Motives and barriers to organic food purchase
Iyer et al., 2016 [38]	Self-administered survey of 251 respondents	Structural equation modeling (SEM)	The role of economic-driven motives, altruistic motives, and price fairness on green product purchase
Marian et al., 2014 [39]	Panel survey of 2512 households in Denmark	Polarization index and Dirichlet method	The role of production method and price on repeat food purchase
Diallo et al., 2015 [40]	A random sample of 671 respondents	Structural equation modeling (SEM)	The relationship among price perception of different food brand types, shopping value dimensions, and store loyalty
Cavaliere et al., [41]	A survey of 306 organic producers and retailers from France, Spain, and Italy	Descriptive statistics	Vertical integration and distribution channels of organic producing companies
Damar-Ladkoo, 2016 [49]	A quota sample of 300 consumers from Mauritius	Descriptive statistics	The effects of guerilla marketing on organic food purchase

## Appendix B

**Table A2:** The results of the estimated logistic regression model for factors that foster organic agriculture products purchase.

Purchase Frequency ^a^		B	Std. Error	Wald	df	Sig.	Exp (B)
1	Intercept	0.355	0.03	140.027778	1	0.254	
	[Healthier than non-organic = 1]	−1.256	0.23	29.8210964	1	0.002	0.285
	[Healthier than non-organic = 5]	0 ^c^			0		
	[Suitable for prevention and treatment of diseases = 1]	−1.125	0.232	23.5141387	1	0.048	0.325
	[Suitable for prevention and treatment of diseases = 5]	0 ^c^			0		
	[Do not contain GMO, pesticides, antibiotics, and other additives = 1]	−0.452	0.078	33.580539	1	0.001	0.636
	[Do not contain GMO, pesticides, antibiotics, and other additive = 5]	0 ^c^			0		
	[Higher nutritional value compared to non-organic = 1]	−0.224	0.078	8.2472058	1	0.035	0.799
	[Higher nutritional value compared to non-organic = 5]	0 ^c^			0		
	[Better taste = 1]	−0.989	0.243	16.564565	1	0.001	0.372
	[Better taste = 5]	0 ^c^			0		
	[Their protection does not harm the environment = 1]	−0.887	0.099	80.27436	1	0	0.412
	[Their protection does not harm the environment = 5]	0 ^c^			0		
	[Organic production ensures animal welfare = 1]	−0.653	0.045	210.57235	1	0.0023	0.520
	[Organic production ensures animal welfare = 5]	0 ^c^			0		
	[Organic products purchase provides support to local producers = 1]	−1.199	0.354	11.4718073	1	0.031	0.301
	[Organic products purchase provides support to local producers = 5]	0 ^c^			0	.	0

Legend: ^a,c^—logit as the link function.

## Appendix C

**Table A3:** The results of the estimated logistic regression model for factors that prevent organic agriculture products purchase.

Purchase Frequency ^a^		B	Std. Error	Wald	df	Sig.	Exp (B)
1	Intercept	21.051	5662.006	0	1	0.997	
	[I do not buy organic products in larger quantities due to high prices compared to my level of income = 1]	0.405	0.056	52.30389031	1	0	1.4993025
	[I do not buy organic products in larger quantities due to high prices compared to my level of income = 5]	0 ^b^			0		
	[I do not buy organic products in larger quantities due to high prices compared to product quality = 1]	0.399	0.067	35.46469147	1	0.003	1.4903336
	[I do not buy organic products in larger quantities due to high prices compared to product quality = 5]	0 ^b^			0		
	[I do not buy organic products in larger quantities because they are not sufficiently represented in stores, so their purchase requires too much time = 1]	0.435	0.066	43.44008	1	0–008	1.544963
	[I do not buy organic products in larger quantities because they are not sufficiently represented in stores, so their purchase requires too much time = 5]	0 ^b^			0		
	[I do not buy organic products in larger quantities because the offerings are scarce (not diversified) = 1]	0.556	0.089	39.02739553	1	0.021	1.7436838
	[I do not buy organic products in larger quantities because the offerings are scarce (not diversified) = 5]	0 ^b^			0		
	[I do not buy organic products in larger quantities because they taste worse than non-organic = 1]	0.205	0.046	19.86059	1	0.049	1.227525
	[I do not buy organic products in larger quantities because they taste worse than non-organic = 5]	0 ^b^			0		
	[I do not buy organic products in larger quantities because I do not know where to find them = 1]	0.243	0.076	10.22316482	1	0.048	1.2750686
	[I do not buy organic products in larger quantities because I do not know where to find them = 5]	0 ^b^			0		
	[I do not buy organic products in larger quantities due to their worse appearance compared to non-organic = 1]	0.307	5.349	0.003294	1	0	1.237385
	[I do not buy organic products in larger quantities due to their worse appearance compared to non-organic = 5]	0 ^b^			0		
	[I do not buy organic products in larger quantities because there are no real differences between organic and non-organic products = 1]	0.112	0.031	13.05306972	1	0.045	1.1185129
	[I do not buy organic products in larger quantities because there are no real differences between organic and non-organic products = 5]	0 ^b^			0		
	[I do not buy organic products in larger quantities because I do not have enough information about their characteristics = 1]	0.298	0.056	28.31760204	1	0.002	1.3471618
	[I do not buy organic products in larger quantities because I do not have enough information about their characteristics = 5]	0 ^b^			0		

Legend: ^a,b^—logit as the link function
